# In Search of Zonation Markers to Identify Liver Functional Disorders

**DOI:** 10.1155/2020/9374896

**Published:** 2020-12-24

**Authors:** Qiuyuan Yang, Shuping Zhang, Juan Ma, Sijin Liu, Shuguang Chen

**Affiliations:** ^1^State Key Laboratory of Environmental Chemistry and Ecotoxicology, Research Center for Eco-Environmental Sciences, Chinese Academy of Sciences, Beijing 100085, China; ^2^University of Chinese Academy of Sciences, Beijing 100049, China; ^3^The First Affiliated Hospital of Shandong First Medical University, Jinan 250014, China; ^4^Department of General Surgery, Peking Union Medical College Hospital, Chinese Academy of Medical Sciences & Peking Union Medical College, Beijing, China

## Abstract

A substantial amount of research is being conducted on zonation markers to identify hepatic injuries and disorders based on the structural and functional zonation of the liver. In contrast to metabolic zonation, hepatocyte ploidy reflects the capability of liver regenerative turnover. Nonetheless, many knowledge gaps remain in the understanding of the links between liver disorders and altered zonation and ploidy, partially owing to the lack of sufficient zonation markers. Under this setting, we recapitulated the currently known and prospective markers used to identify normal and altered liver zonation in different disorders. Furthermore, we discussed new findings from studies that have used advanced methodologies to identify potential markers with greater accuracy. We also elaborated on the perspectives and future applications of zonation research in the early detection of various liver diseases.

## 1. Introduction

**Figure 1:**
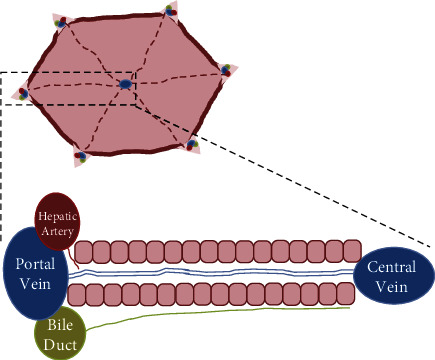
Diagram of the zonal areas in liver lobules. The liver consists of a regular arrangement of hexagonal lobules. At each angle of the lobules, the portal veins, hepatic arterioles, and bile ducts constitute the “portal triads”, whereas central veins are in the center of the lobules.

The concept of liver zonation can be traced back to the 1960s when it was first recognized in mice by Neporent and Glicksman [1]. Since that time, zonation has been studied in liver diseases [2]. With a uniform anatomical structure, the liver consists of hexagonal lobules in the form of a honeycomb. At each angle of the lobules, the portal veins, hepatic arterioles, and bile ducts build up the “portal triads,” whereas central veins are located in the center of the lobules (Figure 1). Hepatocytes are arranged in a spongy manner along the porta-central axis, and their biochemical and physiological functions vary. Six to eight periportal hepatocytes (zone 1) are located in the vicinity of afferent periportal zones, while two to three pericentral hepatocytes (zone 3) are adjacent to the efferent pericentral zones. Zone 2, with six to ten hepatocytes, is located in the middle lobules with unclear boundaries between zones 1 and 3 [3]. All these structural characteristics are termed liver zonation [4].

**Figure 2:**
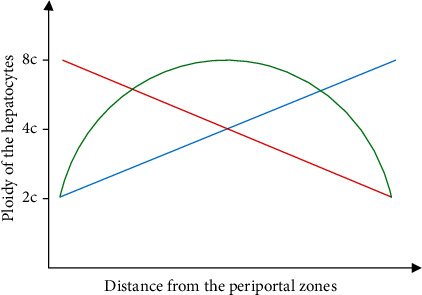
Differential distribution profiles of the hepatocyte ploidy. Although a large number of studies have been performed to shed light on the distribution of hepatocyte ploidy, there are still remarkable controversies to date. Some studies demonstrate that the hepatocytes around the pericentral regions are diploid, different from the polyploid hepatocytes around the periportal regions (red line). However, other reports suggest that multinucleated hepatocytes are adjacent to the pericentral regions (blue line). In the meantime, it has been argued that 4c and 8c polyploid hepatocytes localize in the midzone, rather than in the periportal and pericentral regions (green line).

Liver spatial heterogeneity is manifested by metabolic zonation, as characterized by the varying expression profiles of metabolic genes from the periportal to the pericentral zones, as well as polyploidy of the hepatocytes [5]. In contrast to the distribution of metabolic zonation, hepatocyte polyploidy reflects the capability of liver regenerative turnover [6]. The diploid Axin2^+^ hepatocytes were documented to be more likely the progenitors to fuel polyploid hepatocytes in normal physiology and responses to various stresses [6]. However, other reports also argued that after acute injury, hepatocytes proliferated and regenerated the liver in all regions, in which limited contribution was observed from Axin2^+^ hepatocytes [7]. Moreover, in chronic liver diseases, hepatocyte proliferation was impaired, and instead, the cholangiocytes, located in bile ducts, played a vital role in substituting hepatocytes [8]. In the severely damaged liver, the cholangiocytes were thought to be one of the sources of hepatic progenitor cells, which bore the ability to differentiate into hepatocytes [9, 10]. Previous findings suggested that hepatocytes with different ploidy could be used to distinguish the different zones of the liver. In rodent liver, hepatocytes are diploid with two haploid chromosome sets (2c) at birth, while in adults, most hepatocytes are polyploid, tetraploid (4c), octoploid (8c), or even contain more haploid chromosome sets (e.g., 16c). Nonetheless, the spatial distribution of diploidy and overall polyploidy is still warranted to be studied. Some studies demonstrated that hepatocytes around pericentral zones are diploid, differing from polyploid hepatocytes around the periportal zones [6]. However, there are still some conflicting reports on this aspect, as multinucleated hepatocytes are found next to the pericentral zones [11]. In addition, some studies have argued that 4c and 8c polyploid hepatocytes accumulate in the midlobule zone, rather than in the periportal and pericentral zones (Figure 2) [12]. These inconsistent findings challenge the feasibility of ploidy as a zonation reference.

Genes and biomarkers are being studied as zonation markers to identify liver disorders. Furthermore, more prospective zonation markers are being discovered using new technologies. Nonetheless, more efforts are warranted to improve the poor accuracy and low sensitivity of the currently known liver zonation markers in diagnosing liver injuries and disorders. In the current review, we recapitulated the known and potential markers of liver zonation and assessed their applications and limitations in identifying liver injuries and disorders. Some newly discovered molecules are also discussed as prospective zonation markers to identify liver disorders.

## 2. Current Markers Used to Identify Liver Zonation

### 2.1. The Basis of Functional Zonation of the Liver

**Figure 3:**
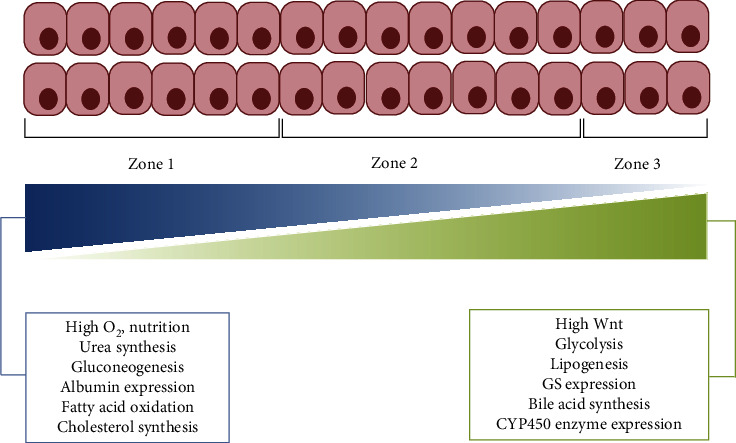
A schematic displays the gradient patterns of basal metabolites across the three zones. The liver cell plate, composed of 15–25 hepatocytes, lies between the periportal and pericentral zones. Six to eight periportal hepatocytes (zone 1) enclose the periportal zone, while two to three pericentral hepatocytes (zone 3) are located near the pericentral zone. The boundary of zone 2, consisting of six to ten hepatocytes, in the middle lobules is still not well-defined. From zone 1 to zone 3, the concentration of O_2_, the synthesis of urea and cholesterol, the gluconeogenesis, the expression of albumin, and the oxidation of fatty acid decrease, whereas Wnt signaling, the expression of glutamine synthetase (GS), and the activity of cytochrome P450 (CYPs) enzyme, the glycolysis, the lipogenesis, and the synthesis of bile acid otherwise increase.

As an essential metabolic organ, the liver exerts functional zonation to efficiently conduct metabolic processes [13]. The portal veins supply the liver with nutrient-rich blood (75% of the total blood supply to the liver), while hepatic arterioles provide the liver with oxygen-rich blood (25% of the total blood supply to the liver). The blood enters the sinusoids and is concentrated in the pericentral zones. The liver cell plate, which is composed of 15–25 hepatocytes, localizes from the periportal to the pericentral zones, and these hepatocytes can be divided into three different zones based on their metabolic activity (zones 1–3). The function of these hepatocytes relies on their location within the liver plate (Figure 3) [3]. Moreover, pericentral regions are more specialized in antioxidative stress and detoxification activities, carried out by glutamine synthetase (GS) and cytochrome P450 (CYP450) enzymes, as well as glycolysis, lipogenesis, and bile acid synthesis. Periportal regions undertake several energy-consuming processes, including cholesterol (CHO) synthesis, urea synthesis, gluconeogenesis, and fatty acid oxidation, and exhibit the highest level of albumin expression (Figure 3) (Table 1) [13, 14].

### 2.2. Markers for Normal Functional Zonation of the Liver

Although CYtP450 enzymes are expressed in all hepatocytes, most xenobiotic metabolism is performed by approximately 50% of the hepatocytes located in the pericentral regions [15]. Moreover, GS, which is expressed in the pericentral regions, catalyzes the condensation of ammonia by converting glutamate to glutamine [16]. Therefore, the expression of CYPs and GS may be a potential marker for the functional zonation of the pericentral regions.

Wnt/*β*-catenin, a master regulator of hepatocyte proliferation and liver metabolic zonation, is activated in the pericentral zones. Adenomatous polyposis bacillus, a negative regulator of Wnt signaling, is absent in the periportal regions and is recognized as a zonation-keeper of the liver [3]. Progenitor cells are widely identified by their preferential expression of progenitor cell markers, such as *Axin2* and *Tbx3*. The expression of *Axin2*, a transcriptional target gene downstream of Wnt/*β*-catenin signaling, offers a reliable readout of the response of hepatocytes to Wnt signaling. Moreover, the Wnt protein acts as a transient signal that maintains the pluripotency of stem cells in the liver, as *Tbx3*, another target gene downstream of Wnt signaling, is identified as a stem cell marker expressed by pericentral hepatocytes [6]. Thus, *Axin2* and *Tbx3* could be used as functional zonation markers for the pericentral regions in order to maintain liver topology (Table 2).

In the periportal regions, hepatocytes express a high level of arginase and carbamoyl phosphate synthetase-1 (CPS1) to carry out metabolic functions. Arginase converts L-arginine to L-ornithine and urea, and thus affects the activity of hepatic nitric oxide synthase by reducing L-arginine bioavailability. CPS1, an ATP-dependent enzyme, converts ammonia and carbon dioxide to carbamoyl phosphate [17]. Thus, both arginase and CPS1 could serve as functional zonation markers of the periportal regions. Major facilitator superfamily domain containing 2a (*Mfsd2a*), which was previously recognized as a protein that maintains the blood-brain barrier, could also be a functional zonation marker of the periportal zones (Table 2) [18].

## 3. Disordered Liver Zonation Points to Abnormal Metabolism and Potential Risks of Morbidity

The great potential of liver zonation resides in its potential to aid the diagnosis of abnormal liver metabolism and risks for various diseases. In 1996, Lorraine et al. investigated zonal metabolic enzymes and found that marked metabolic zonation of normal lobules was retained in fibrotic hepatic lobules in humans; however, this zonation pattern was lost in cirrhotic lobules [19]. Although diverse liver disorders and injuries may alter zonation, reports on diagnosis using zonation markers are still limited to only a few liver diseases and injuries, including nonalcoholic fatty liver disease (NAFLD), nonalcoholic steatohepatitis (NASH), and hepatocellular carcinoma (HCC), as discussed below.

### 3.1. Hepatic Metabolic Diseases

The liver is the main organ for the metabolism of fats, carbohydrates, urea, bilirubin, and vitamins [20]. Thus, biochemical indicators are closely involved in the metabolic processes in the liver and are frequently used as markers to identify liver zonation. With an increasing number of cases of hepatic metabolic diseases that cause severe damage to liver functions and even systemic impairments, fundamental studies on markers are necessary to determine the hepatic metabolic zonation profiles in both healthy and diseased conditions. For example, canonical Wnt/*β*-catenin and hypoxia-inducible factor signaling pathways were found to be connected with altered liver zonation together with the pathogenesis of hepatic metabolic diseases [21, 22]. Lipogenesis mainly occurs in pericentral hepatocytes, while fatty acid oxidation occurs in periportal hepatocytes [23]. Furthermore, fatty acid uptake is greater in periportal regions, and the expression of fatty acid-binding protein tends to decline from the periportal to pericentral zones. Together, these findings suggest that the genes and biomarkers involved in fatty acid metabolism could be prospective zonation markers for identifying hepatic metabolic diseases [24].

NAFLD, which is present in 25% of the world's population, starts with the uncontrolled accumulation of fat in hepatocytes, causing inflammation and fibrosis in the liver [25]. In NAFLD patients, hepatic steatosis is obvious in pericentral hepatocytes [14]. A high-fat diet was found to induce the deposition of triglycerides (TGs) in areas surrounding the periportal zones in NAFLD animal models, suggesting that TGs might be a marker to identify NAFLD (Table 3) [26]. Different types of high-fat diets (e.g., solid vs. liquid) might result in divergent deposition patterns of TGs, which is indicative of different zonation patterns [27]. NASH frequently causes hepatic fibrosis by inducing hepatocyte injury and inflammatory cell infiltration. Although the level of serum bile acids is elevated, and the lysophosphatidylcholine level is reduced, in NASH patients, the liver zonation changes in NASH patients, and their contribution to NASH remain largely unknown [28]. The enrichment of arachidonic acid (AA) in pericentral hepatocytes leads to enhanced oxidative stress and consequent damage to pericentral regions in mice with NASH [29]. Additionally, the spatial distribution of the remodeling enzyme, lysophosphatidylcholine acetyltransferase 2, plays a role in changing the zonal location of AA-containing lipids [29]. Thus, AA-containing lipids might be used as markers for predicting the risk of zonal NASH. However, there is still no reported zonation marker to determine altered glycolysis and gluconeogenesis under hepatic metabolic diseases.

### 3.2. Hepatocellular Carcinoma (HCC)

HCC is a highly prevalent cancer and is the third leading cause of death among cancers [21]. Unfortunately, limited therapeutic choices are available for patients with advanced HCC, yielding an urgent demand for the early detection and diagnosis of this disease [30]. Alpha-fetoprotein (AFP) is a known marker for HCC, and its levels are elevated up to 70% in the sera of HCC patients. Thus far, AFP has been widely used as an adjuvant diagnostic index. Nonetheless, the pathophysiological knowledge of AFP expression in different liver zones remains elusive [30]. Similarly, neuropilin 1, a transmembrane glycoprotein with increased expression in HCC, is also a novel HCC marker [31]. Although considerable progress has been made in identifying biomarkers for HCC, little insight has been gained on their likely zonated expression in HCC patients. Intriguingly, HCC manifests a high predilection of invasion towards portal veins, leading to an extremely poor prognosis [32]. Portal veins are located near the bile ducts. To this end, HCC patients with a bile duct thrombus display differential progression of HCC, including a higher rate of lymphovascular invasion, macrovascular invasion, and poor differentiation [33].

The Wnt signaling pathway, which is more active in pericentral liver regions, is closely implicated in HCC. Wnt receptors (e.g., frizzled class receptor 7 (Fzd7) and Fzd8) are pericentrally zonated. In contrast, Wnt inhibitors (e.g., catenin beta interacting protein 1 and transcription factor 7-like 1 (Tcf7l1)) are periportally zonated [34]. Moreover, microRNAs (miRNAs), which are also differentially expressed among zones in the liver, contribute to the zonated activity of Wnt signaling. For example, the pericentrally upregulated expression of miR-93 inhibits Tcf7l1 expression, leading to increased Wnt signaling activity in the pericentral zones. In contrast, the periportally downregulated expression of miR-99a and miR-100 leads to a loss in their capability to inhibit the expression of Fzd8, and thus enhances Wnt activity in the periportal zones [34]. Under this setting, the expression patterns of these miRNAs might be used as zonation markers to detect early HCC.

### 3.3. Liver Injuries following Toxin Exposure

Exposure to chemicals and even particulate matter can give rise to liver impairments. Along with the increasing understanding of injury-related zonation, some markers have been suggested to predict the risk of liver injury. For example, the accumulation of copper and iron is graded by location (pericentral, midzonal, and periportal zones) [35]. From the pericentral to the periportal zones, the concentration of iron gradually decreases, while the concentration of copper increases [36]. Moreover, the zonated accumulation of some metals, particularly copper and iron, between the periportal and pericentral zones, is altered, and even reversed, in rats upon exposure to 3,3′,4,4′,5-pentachlorobiphenyl [36], suggesting that copper and iron zonation might be potential indicators for detecting the risk of liver injury induced by chemicals.

Other toxic xenobiotics, such as CCl_4_, an established chemical to induce animal models of hepatic fibrosis, are metabolically activated by CYPs, such as CYP1A, CYP2C, CYP2E1, and CYP3A, and cause hepatocyte necrosis in a pericentral pattern [15]. Acetaminophen intoxication has also been shown to cause necrosis of pericentral hepatocytes [34]. Therefore, CYPs might be used as a potential indicator of pathological changes in the pericentral regions [15]. Moreover, miRNAs are stable in circulation and could be potential biomarkers to indicate the risk of hepatic injuries. A retrospective study revealed that most pericentral miRNAs were enriched in plasma, but not in the periportal zones in acetaminophen-treated mice [34]. Additionally, L-arginine treatment induced a clear zonation of liver injury, as characterized by congested sites around pericentral zones and invasion of inflammatory cells around periportal zones [37]. In addition to metabolic functions, the localization of toxins is also crucial in determining their zonated toxicity. For instance, graphene oxide changes the expression levels of some proteins, such as E-cadherin, due to its preferential accumulation in the periportal zones [38]. Therefore, different toxins may elicit distinct effects on liver zonation, which makes it difficult to obtain universal zonation markers to detect liver injuries for all toxins. Therefore, both universal and specific markers are required.

## 4. Continued Efforts in Search of New Zonation Markers

Although some biomarkers and genes have been reported as markers for identifying liver zonation in both normal and pathological states, issues in the early detection of liver disorders have not been addressed. To overcome the technical challenges, some emerging strategies and techniques, such as single-cell RNA sequencing (scRNA-seq), are being employed to shed light on the heterogeneity of hepatocytes and disease-related cellular reprogramming [14]. In combination with single-molecule fluorescence in situ hybridization, scRNA-seq can generate a high-resolution visual map of RNA expression in spatial and global dimensions of the liver, making it feasible to explore more accurate zonation markers in the future [39].

With the aid of scRNA-seq technology, which helped determine hepatic transcriptomes at the single-cell level, hepatocytes and liver sinusoidal endothelial cells (LSECs) were identified to reveal marked features of zonation in mouse and human livers [40, 41]. The zonation profiles for hepatocytes are shown below (Table 3). By analyzing hepatocyte-characterized zonation, some genes, such as *ALB* and *PCK1*, are located in periportal zones, while *GLUL* is expressed in pericentral zones. By analyzing LSEC-characterized zonation, 806 genes (out of a total of 1198 genes) were found to have zonal expression profiles, including *ICAM1* and *ENG* (central region), and *BTNL9*, *ANPEP*, *MTHFS*, and *GATM* (periportal region). Lymphatic vessel endothelial hyaluronan receptor 1 and CD14, which have been validated at the protein level, are recognized as markers to distinguish between central and midzonal LSECs [42]. Thus, zonal protein profiles of these genes might be further developed as prospective markers to distinguish the different zones of the liver (Table 3) [40].

## 5. Concluding Remarks and Perspectives

Liver zonation is important for the physiology of the liver, but disordered zonation is also closely associated with diverse liver diseases. Liver zonation is currently defined through zonation markers, such as biochemical indicators and genes. However, these zonation markers are subject to change under various pathological conditions, and the changes in these markers, in turn, could be used to recognize liver disorders and injuries, especially at the early stage. Despite recent encouraging progress, more selective zonation markers with higher accuracy are needed for early diagnosis.

In general, more work is required in the study of zonation markers. First, specific zonation markers have not yet been identified to represent certain liver disorders, and extensive work is needed to find accurate zonation markers for the early detection of liver disorders. Second, new markers, or marker toolboxes, should be identified with the aid of advanced biotechnologies, such as scRNA-seq. With these new markers, more information would be obtained to elucidate liver metabolism under normal physiological and diseased conditions. Third, given biomarkers for liver regeneration and progenitor cells, a new opportunity to decipher the mechanism(s) underlying the formation of liver zonation would be possible. Therefore, additional work to determine the molecular bases for self-renewal regulation is warranted. Finally, current methods to identify liver zonation mainly rely on immunohistochemical staining, the polymerase chain reaction, and other sophisticated technologies. However, these methods involve difficult and expensive experimental procedures. Thus, simplified readouts should be developed for the detection of altered liver zonation, such as serum biochemical markers, which will be insightful in detecting changes in liver zonation through conventional examinations.

## Figures and Tables

**Table 1:** Current biochemical indicators used as markers to identify liver zonation.

Zone	Biological processes	Biochemical indicators	Detection methods	References
Pericentral zones	Glutamine synthetase expression	Glutamine synthetase	Glutamine synthetase assay	[13]
	Cytochrome P450 (CYPs) enzyme expression	CYP450	CYP450 ELISA	
	Bile acid synthesis	Bile acids	Total bile acid assay	
	Lipogenesis	Acetyl-CoA carboxylase	Acetyl-CoA carboxylase assay and western blot	[23]
		ATP citrate lyase	ATP citrate lyase assay	
	Glycolysis	Glycolytic enzymes	Protein levels of hexokinase II and lactate dehydrogenase-A	[43]
		Lactate	Lactic acid assay	
		Lactate dehydrogenase	Lactate dehydrogenase assay	

Periportal zones	Cholesterol synthesis	Cholesterol	Total cholesterol assay	[13]
	Urea synthesis	Urea	Urea assay	
	Albumin expression	Albumin	Albumin assay	[14]
	Fatty acid oxidation	Carnitine palmitoyltransferase-1	Carnitine palmitoyltransferase-1 assay	[23]
	Gluconeogenesis	Gluconeogenic genes and protein expression	qRT-PCR and western blot	
		Glucose	Blood glucose monitor	

**Table 2:** Current genes used as markers to identify liver zonation.

Zone	Gene symbol	Biological function	Detection methods	Sources	References
Pericentral zones	*Cyp1a*	CYPs serve to detoxify xenobiotics prior to being drained into the pericentral zone.	Frozen section + immunostaining	Rat anti-CYP1A2 antibody	[15] [44]
	*Cyp3a*			Rabbit anti-CYP3A1 antibody, Biotrend, Germany	
	*Cyp2c*			Rat anti-CYP2C6 antibody	
	*Cyp2e1*		Frozen/FFPE section + immunostaining	Rabbit anti-CYP2E1 antibody, Sigma-Aldrich, USA	
	*Gs*	GS catalyzes the condensation of ammonia by converting glutamate to glutamine.	Frozen section + immunostaining	Mouse anti-GS antibody, BD Bioscience, Germany RNAscope probes: NM 008131, region 103-973 Anti-GS (clone 2B12) antibody, Sigma-Aldrich, USA	[16]
			In situ hybridization		
			Western blot		
			qRT-PCR		
	*Axin2*	A universal transcriptional target of Wnt/*β*-catenin signaling is *Axin2*, whose expression offers a reliable readout of hepatocytes responding to Wnt.	In situ hybridization	RNAscope probes: NM 015732, region 330-1287 Chicken anti-GFP antibody, Abcam ab13970, UK	[6]
			Frozen section + immunostaining (GFP-tag model)		
	*Tbx3*	*Tbx3* is another target gene of Wnt signaling, which has been identified as a progenitor marker of hepatocytes.	In situ hybridization	RNAscope probes: NM 198052	

Periportal zones	*Arg1*	Arginase converts L-arginine to L-ornithine and urea, affecting hepatic nitric oxide synthase (NOS) activities by decreasing L-arginine bioavailability.	Frozen section + immunostaining Western blot qRT-PCR	Rabbit anti-arginase-1 antibody, Sigma-Aldrich, USA Anti-arginase-1 antibody, BD Transduction Laboratories, CA	[17]
	*Cps1*	CPS is an ATP-dependent enzyme and converts ammonia and carbon dioxide (CO2) to carbamoyl phosphate.	Frozen section + immunostaining qRT-PCR	Rabbit anti-CPS1 antibody, Abcam, UK	
	*Mfsd2α*	Mfsd2a, previously recognized as a protein that maintains blood-brain barrier function, is a periportal zonation marker.	Frozen section + immunostaining (RFP-tag model)	Rabbit anti-RFP antibody, Rockland 600-401-379, USA	[18]

Note: *Cyp*: cytochrome P450; *Gs*: glutamine synthetase; *Tbx3*: T-box transcription factor 3; *Arg1*: Arginase1; *Cps1*: carbamoyl phosphate synthase 1; *Mfsd2a*: major facilitator superfamily domain-containing 2a; FFPE: formalin-fixed paraffin-embedded.

**Table 3:** Newly-defined molecules as prospective markers to identify liver zonation.

Zone	Potential markers	Biological processes/function	Detection methods	References
Pericentral zone	*ICAM1*	This gene encodes cell surface glycoproteins mainly expressed on endothelial cells.	Immunostaining (protein) Diffusion pseudotime (DPT) analysis and self-organizing maps (SOMs) (gene)	[40] [45]
	*GLUL*	The protein encoded by this gene plays a role in acid-base homeostasis, ammonia and glutamate detoxification, and cell proliferation.	Immunostaining (protein) DPT and SOMs (gene)	
	*ENG*	This gene encodes a transmembrane protein, which is a glycoprotein of the vascular endothelium.	Immunostaining (protein) DPT and SOMs (gene)	
	miR-93	miR93 inhibits the expression of transcription factor 7-like 1 (Tcf7l1).	qRT-PCR	[34]

Periportal zone	*ALB*	The protein encoded by this gene plays a role in the regulation of blood plasma colloid osmotic pressure and acts as a carrier protein.	DPT and SOMs (gene)	[40] [45]
	*BTNL9*	The expression of the protein encoded by this gene is found in several types of cancers.	DPT and SOMs (gene)	[40]
	*ANPEP*	The protein encoded by this gene is expressed in plasma membranes.	Immunostaining (protein) DPT and SOMs (gene)	
	*PCK1*	This gene is the main control point for the regulation of gluconeogenesis.	Immunostaining (protein) DPT and SOMs (gene)	[40] [45] [46]
	*MTHFS*	The protein encoded by this gene is an enzyme that catalyzes the conversion of 5-formyltetrahydrofolate to 5,10-methenyltetrahydrofolate.	Immunostaining (protein) DPT and SOMs (gene)	[40] [46]
	*GATM*	This gene encodes a mitochondrial enzyme that is involved in creatine biosynthesis.	Immunostaining (protein) DPT and SOMs (gene)	
	Liver fatty acid-binding protein (L-FABP)	This protein controls fatty acid uptake.	Immunostaining	[24]
	Triglyceride (TG) (high-fat diet (HFD)-induced nonalcoholic fatty liver disease (NAFLD) mouse model)	TG is the composition of lipids.	TG assay (liver and plasma)	[26]
	miR-99a and miR-100	They inhibit the expression of frizzled class receptor 8 (Fzd8).	qRT-PCR	[34]

Note: *ICAM1*: intercellular adhesion molecule 1; *GLUL*: glutamate-ammonia ligase; *ENG*: endoglin; *ALB*: albumin; *BTNL9*: butyrophilin 9; *ANPEP*: alanyl aminopeptidase; *PCK1*: phosphoenolpyruvate carboxykinase 1; *MTHFS*: methenyltetrahydrofolate synthetase; *GATM*: glycine amidinotransferase.

## Data Availability

No data were used to support the study.
